# Chromosome-Level Haplotype Assembly for *Equus asinu*

**DOI:** 10.3389/fgene.2022.738105

**Published:** 2022-05-27

**Authors:** Xinyao Miao, Yonghan Yu, Zicheng Zhao, Yinan Wang, Xiaobo Qian, Yonghui Wang, Shengbin Li, Changfa Wang

**Affiliations:** ^1^ Liaocheng Research Institute of Donkey High-Efficiency Breeding and Ecological Feeding, Liaocheng University, Liaocheng, China; ^2^ Department of Computer Science, City University of Hong Kong, Hong Kong, Hong Kong SAR, China; ^3^ College of Forensic & Medicine, Xi’an Jiaotong University, Xi’an, China; ^4^ Shenzhen Byoryn Technology Co., Ltd., Shenzhen, China

**Keywords:** donkey, *Equus*, haplotype, population analysis, phase

## Abstract

**Background:** Haplotype provides significant insights into understanding genomes at both individual and population levels. However, research on many non-model organisms is still based on independent genetic variations due to the lack of haplotype.

**Results:** We conducted haplotype assembling for *Equus asinu*, a non-model organism that plays a vital role in human civilization. We described the hybrid single individual assembled haplotype of the Dezhou donkey based on the high-depth sequencing data from single-molecule real-time sequencing (×30), Illumina short-read sequencing (×211), and high-throughput chromosome conformation capture (×56). We assembled a near-complete haplotype for the high-depth sequenced Dezhou donkey individual and a phased cohort for the resequencing data of the donkey population.

**Conclusion:** Here, we described the complete chromosome-scale haplotype of the Dezhou donkey with more than a 99.7% phase rate. We further phased a cohort of 156 donkeys to form a donkey haplotype dataset with more than 39 million genetic variations.

## 1 Introduction

One of the domesticated members of *Equidae*, the donkey (*Equus asinu*), since its domestication in tropical and subtropical Africa about 7,000–9,000 years ago, has provided transportation, fertilizer, and food for humans (Smith and Pearson, 2005; Han et al., 2014; Wang et al., 2020). Domesticated donkeys are the primary livestock for farming and transport in Africa (Nengomasha et al., 1999a; Nengomasha et al., 1999b; Geiger and Hovorka, 2015) and a highly nutritious food source in Asia (Stat, 2020). As of 12 November 2020, there are more than 50 million asses worldwide (Stat, 2020); however, the number is dramatically decreasing in Europe and Asia, while increasing in Africa due to the human lifestyle shifting. Moreover, donkeys have been perceived as disease-resistant, drought-resistant, and hardy species (Swai and Bwanga, 2008). Donkeys also provide insights for medical research and the potential as medical model animals. For instance, donkey milk could be a surrogate for breast milk (Souroullas et al., 2018; Conte and Panebianco, 2019) and a possible treatment for type 2 diabetes, colitis, and breast cancer; donkey serum albumin hydrolysates can potentially inhibit tumor cell proliferation (Li et al., 2013; Jiang et al., 2018; Kim et al., 2018; Li et al., 2020).

Although donkeys were preferred to other *equines* because of their affordability, survivability, and medical and economic value, the lack of haplotype databases has limited genetic improvement or manipulation. More and more chromosome-scale assemblies for haplotype analysis have been published, even for the non-model organisms, such as laboratory mice and heterozygous diploid potatoes (Mott, 2007; Huang et al., 2012; Zhou et al., 2020). With resequencing, haplotype information may reveal recombination rate, genome variation, *de novo* mutation, and the selection efficiency among the population, providing information on the population structure and the evolutionary history (Stapley et al., 2017). Phasing, which refers to the process of assembling haplotypes, offers significant insights into the understanding and detection of many genetic variations (Yen et al., 2020). A chromosome-scale and haplotype-phased genome can provide a complete gene repertoire, a richer set of linkage information, a higher precision on the location, number, and functional genes of QTLs, the genetic architecture of the traits, and the molecular mechanisms underpinning trait variation (Leitwein et al., 2020).

There are two copies of autosomes for diploid organisms, namely, the paternal and maternal copies. Haplotype information consists of a set of DNA variations in a specific sequence on each homologous chromosome copy. Phasing, known as haplotype reconstruction, is essential for population demography, biological conservation, and hereditary diseases (Stephens et al., 2001; Danecek and McCarthy, 2017; Hui et al., 2017). Haplotypes are inferred from single individual genome sequence data, linkage analysis of population data, or trio-data (Bekele et al., 2018). Many bioinformatics tools have been developed for phasing, mainly divided into indirect and direct approaches. 1) Indirect approaches use the genetic information of related or unrelated individuals to infer haplotypes. By using unrelated individual genotypes, the population-based phase indirectly reconstructs the haplotype from population reference panels (Loh et al., 2016). 2) Direct approaches are mainly based on the second- or third-generation sequencing data to perform haplotype phasing of single individuals (Bansal and Bafna, 2008). 3) The trio phase is based on Mendel’s law, which is a combination of indirect and direct methods (Garg et al., 2016).

Herein, based on the available data and the previously assembled genome of *Equus asinu*, we presented a chromosome-level haplotype of the Dezhou donkey genome, with the high-depth sequencing data from diverse strategies, including Pacific BioSciences single-molecule real-time sequencing (PacBio SMRT), paired-end next-generation Sequencing (NGS, Illumina reads), and Hi-C. The Dezhou donkey is one of China’s giant local breeds. Advancements in sequencing technologies have promoted variant discovery, genotyping, and phasing (Ellegren, 2014). NGS enables accurate genome assembling, while third-generation sequencing (TGS) fundamentally improves the continuity and completeness of the assembly (Jayakumar and Sakakibara, 2019). Moreover, Hi-C sequencing, which captures genome-wide chromatin interactions, provides further information for anchoring the genomic data to chromosomes (Akgol Oksuz et al., 2021). Here, we extended our software package SpecHap to resolve the phasing of different sources of sequencing data (Yu et al., 2021) and applied it to the donkey. We obtained a haplotype reference panel based on *Equus asinu* population for integrating read-based and population-based phasing results.

## 2 Materials and Methods

**FIGURE 1:**
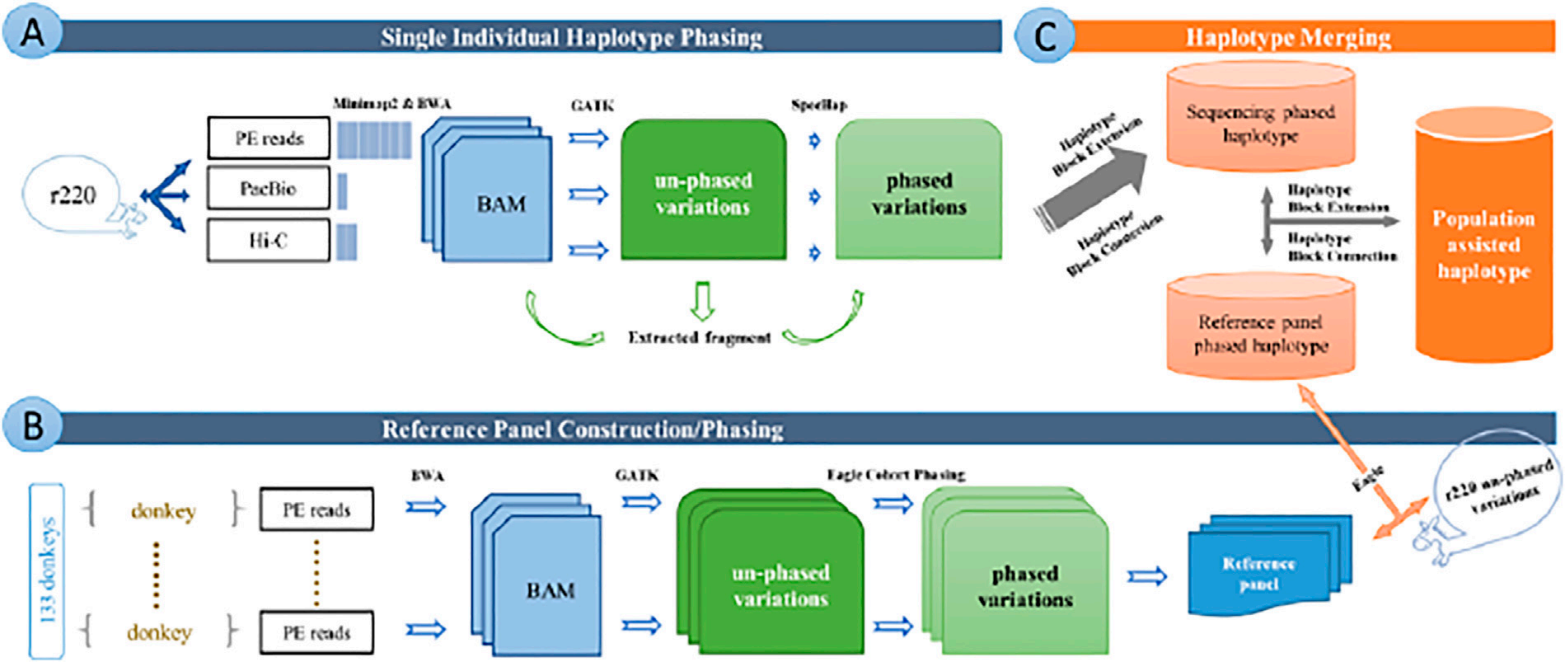
Overview of the whole pipeline of data editing and analyzing. **(A)** Pipeline of single individual haplotype phasing. **(B)** Pipeline of reference panel construction and cohort phasing. **(C)** Pipeline of haplotype merging. Figures show the all working procedure for chromosome-level haplotype construction from sequencing data.

We summarized the working procedure for chromosome-level haplotype construction from the sequencing data in Figure 1.

### 2.1 Sequencing Data Generation and Pre-Processing

#### 2.1.1 Sequencing Data of the 156-Donkey Cohort

First, we sequenced the 23 Dezhou donkey jack data, forming a part of the 156-donkey cohort from the Donkey Research Institute (Dong'E County, Shandong Province, China). We collected blood samples from 23 domestic donkeys from Dezhou city in China. Then we adopted Illumina NovaSeq to conduct NGS sequencing. The average sequencing reads of the 23 donkeys we sequenced was 212,775,969.6, and the average number of bases was 15,958,197,717.

Second, we downloaded the next-generation sequencing data of 133 donkey individuals. 1) Illumina reads resequencing data of 85 individuals were downloaded, based on Illumina HiSeq 4000, with sequencing depth ranging from ×7 to ×50 (PRJNA431818). 2) We downloaded the other 48 donkeys’ resequencing data (KT896508-KT896510, SRR1562345, ERR650932-ERR654612, ERR669419-ERR669469, ERR650540-ERR650547, ERR650570-ERR650703, and PRJCA001131).

Animal care and research procedures were carried out following the guiding principles for the care and use of laboratory animals. The Animal Welfare & Ethics Committee approved all animal protocols of the Institute of Animal Sciences, Liaocheng University (No. LC 2019-1).

#### 2.1.2 Sequencing Data of Individual Donkeys

Here, we used two individual donkeys for haplotype phasing. 1) One donkey individual, r220, has data generated from multiple sequencing strategies, including Illumina short-read sequencing, PacBio SMRT sequencing (traditional low-accuracy PacBio reads), and Hi-C sequencing, in the GenBank database under accession SRS3193390. The NGS data was sequenced on both short-insert and mate-pair libraries with insertions ranging from 170 bp to 40 kbp, generating around ×211 raw data. The PacBio SMRT sequencing was prepared with a 20 kb insert size SMRTbell library, collected around ×30 raw reads and estimated based on the diploid genome. We processed Hi-C data following the protocol described by Rao et al. The sequencing yield was about ×56, estimated based on the donkey genome (Rao et al., 2014). 2) We also utilized the high-depth resequencing data from another diploid donkey jack individual, Willy, with an average depth of coverage of ∼×60, based on Illumina HiSeq platforms with the Chicago HiRise library (Renaud et al., 2018).

#### 2.1.3 Sequencing Data Pre-Processing

We applied the following criteria to filter reads from both short insert and extensive insert libraries to obtain high quality data. First, reads in which the proportion of N exceeds 2% were filtered out. Then, we dropped the reads in the following categories: low quality, adapter contamination, insert size abnormal, or PCR duplication. 1) The reads were marked as low quality if 40% of bases had quality scores ≤7. 2) Adapter contamination was detected if the reads were aligned to the adapter sequence with more than 10 bp overlap (mismatches ≤3 bp). 3) Abnormal insert size was marked if the overlap length of the reads pair was ≥10 bp (10% mismatches) or when the length of the reads pair was minor, then the insert size <30. 4) Duplications were determined when two reads’ pairs are identical.

We aligned the filtered reads to the *Equus asinu* reference GCA_016077325.1 (Wang et al., 2020). The alignments were performed with bwa-mem version 0.7.17 with default alignment parameters (Li and Durbin, 2009). As for the high-depth sampled donkey individual, the Illumina reads were aligned with bwa-mem. Hi-C reads were aligned with bwa-mem with option −5SP specified. PacBio SMRT reads were aligned with minimap2 with preset parameters for PacBio alignment (Li, 2018). We marked the duplications with Picard MarkDuplicates (version 2.1). Then, we performed the genotyping and genetic variations with GATK HaplotypeCaller version 4.0 with default parameters.

### 2.2 Phasing of Individual Donkey Genome

We adopted our recently published software, SpecHap (Yu et al., 2021), which uses spectral analysis to realize genome-wide individual haplotyping in diploid for various sequencing protocols, as presented in Figure 1A.

1) First, we utilized the extractHAIR software bundled with SpecHap for fragment extraction from sequence alignment and the high-quality set of variant genotypes with NGS data.

2) Then, SpecHap divided the chromosomes into sliding windows. We adopted the default sliding windows size, which is 200 variation loci.

3) We extracted the linkage information and constructed internal graphs for different platform sequencing data.

4) Next, we calculated the unnormalized graph Laplacian based on the adjacency matrix.

5) Finally, we obtained two haplotype strings produced by the Fielder vector. We assigned phased variants and outputted them in VCF format.

For Hi-C data, we filtered the fragments if the insert size was greater than 40 Mbp to avoid possible phasing error introduced by trans interaction between homologous chromosomes. As for the PacBio SMRT data with a higher sequencing error rate, realignment was performed with extractHAIR according to the donkey’s reference with minimum base quality to consider a base for haplotype fragment set to 20.

### 2.3 Donkey Population-Assisted Phasing and Haplotype Extension

We constructed a donkey haplotype reference panel to improve haplotype inference and build a variation database of *Equus asinu* population haplotype, as displayed in Figure 1B. The merged individual haplotype assembly was further extended with sequencing data from the population-inferred haplotype reference panel.

We used 156 donkeys to perform cohort phasing according to identity by descent with Eagle v2.4.1 (Hui et al., 2017) with a built-in linear genetic map. We adopted the phased result as the donkey haplotype reference panel and further phased 156 donkey individuals with Eagle reference-panel phasing mode. In addition, the phased individual haplotype assembly was further extended with a population-inferred haplotype reference panel. We adopted Eagle with an experimental feature usePS (use Phase Set) set to true.

Then, we developed an in-house script to merge the haplotype block from single individual phasing and reference panel phasing (https://github.com/yonghanyu/DonkeyHaplotype; Figure 1C). Our script requires two sets of phased VCF files as input with different priorities. It then constructs a connection graph with nodes representing haplotype blocks. Nodes from the high-priority haplotype block were marked as primary, while nodes from the low-priority were marked as secondary.

Edge is added if two connected haplotype blocks share more than N phased SNVs, N is defined by the user. There will be two situations: a secondary node connected to multiple primary nodes, or a primary node connects to a secondary node with a degree equal to 1. Our script connects the corresponding high-priority haplotype blocks depending on the first case’s low priority haplotype block. For the other case, it leads to haplotype extension given a pair of high-priority and low-priority blocks. The detailed haplotype merging algorithm is outlined in Algorithm 1.

### 2.4 Estimation of Recombination Rate, Linkage Disequilibrium, and Effective Population Size

We further estimated the recombination rate of variations based on the haplotype of 156 donkey individuals. The bcftools consensus was adopted to convert the population into a fasta format. We then utilized LDJump (Hermann et al., 2019) to estimate the recombination rate for 30 autosomes. We evaluated the recombination rate for a fixed-size genomic segment of 1000 bp for whole-genome estimation and 100 bp for individual genes. We estimated the recombination rate for segments with less than two single-nucleotide polymorphisms (SNPs) *via* imputation.

For LD decay analysis, we employed PopLDdecay (Zhang et al., 2019) to estimate the squared Pearson correlation (r^2^), based on variant call format (VCF), for each chromosome. In addition, we set r^2^ as 0.5 when calculating the LD decay distance because it exceeds 200 kb when r^2^ is 0.1.

For effective population size (*Ne*), we used SMC++ to infer donkey demography, mainly relying on the unphased data (Terhorst et al., 2017). We obtained phased and unphased variations from a single donkey, r220. We used the ‘smc++ vcf2smc–d r220’ according to the optional arguments for unphased data to specify a single individual donkey. Subsequently, we selected a small cohort of 14 donkeys from 156 donkey groups based on the following conditions (see Supplementary Table S1). 1) We selected five donkeys from China based on the geographic locations, including Dezhou, Yunnan Province, Biyang County, Xinjiang Province, and Tibet Province. 2) We selected five donkeys from all over the world, including Kenya, Egypt, Nigeria, Iran, and Ethiopia. 3) We selected four different species of wild donkeys, including *Equus hemionus*, *Equus kiang*, *Equus hemionus onager*, and *Equus africanus somaliensis*. We obtained phased and unphased variations from 14 donkeys. We generated phased variations from 14 donkeys based on two reference panels of different sizes, 156 donkeys and 14 donkeys, respectively. We used the “smc++ vcf2smc–d r220” according to the optional arguments for unphased data to specify different donkey populations.

We used three databases: PubMed, Web of Science, and Google Scholar, to conduct a systematic search for studies on the association between donkeys and diseases or traits published from 2017 to December 2020. We focused on the studies with transcriptome sequencing and RT-PCR verification results. Based on these conditions, we selected four genes for recombination rate and LD within a smaller window size, *TBX3*, *TEP1*, *MSTN*, and *KITLG*, which played an essential role in livestock. KITLG regulates spermatogenesis in the donkey genome and has possible consequences on speciation and reproductive isolation (Renaud et al., 2018). Exposed to natural contamination, genes *PTEN*, alias *TPE1*, are related to the PTEN signaling pathway related to donkeys’ ovarian cancer (Zhang et al., 2018). Another study showed that the decrease in the expression of *TBX3* in donkeys has an inhibitory effect on pigmentation. MSTN is involved in donkeys’ growth and skeletal development (Liu et al., 2017).

## 3 Results

### 3.1 Pseudodiploid Chromosome Genome of *Equus asinu*

We first assembled and evaluated haplotypes from three sequencing data types for a single donkey (r220), paired-end Illumina reads sequencing, PacBio SMRT sequencing, and Hi-C sequencing. The set of variants that we adopted for phasing was genotyped with NGS data. Here, we utilized VCF as a standardized format for storing the haplotypes.

Although with a higher per-base error rate, the PacBio SMRT assembled haplotype could achieve comparable accuracy since most errors introduced during phasing are caused by fragment duplication. For Hi-C data that introduces trans-interaction errors between homologous chromosomes, high-quality phased blocks were acquired by filtering read pairs with an insert size greater than 40 Mbps. Due to the large span, we assessed one block with the most heterozygous variants phased (MVP) in Hi-C data (Table 1). We utilized paired-end Illumina reads, PacBio, and Hi-C sequencing data to construct sequencing phased haplotype separately.

**TABLE 1:** Haplotype block statistics with diverse sequencing protocols for r220.

	Adjusted N50/span^a^	N50/span	Phased SNVs	Phased SNVs (percent)
Paired-end Illumina reads	NA	761	1,813,095	78.44%
PacBio	44,600	38,045	2,182,637	94.42%
Hi-C	10,399	144,465,297	977,598	42.30%
Hi-C (only for MVP block)	6,430,076	228,685,866	6,005	NA
Sequencing phased haplotype^b^	87,806,226	98,177,835	2,283,521	98.79%
Population-assisted haplotype^c^	93,063,389	98,511,941	2,305,298	99.73%

a AN50 is defined as N50 of adjusted span, which is the span of haplotype block times the proportion of phased SNP.

b Sequencing phased haplotype was phased based on merged sequencing data from multiple protocols: Illumina reads, PacBio, and Hi-C.

c Population-assisted haplotype is a combination of sequencing phased haplotype and reference panel-based haplotype.

As displayed in Table 1, we measured the continuity of haplotype statistics by the Adjusted N50 (AN50), N50, the number of phased SNVs, and phase rates. The AN50 is defined as N50 of adjusted span, the span of haplotype block times the proportion of phased SNP (Yu et al., 2021). The reads from different sequencing protocols were merged into a pool to perform hybrid assembly with SpecHap–hybrid set. With Illumina reads, 78.44% of heterozygous SNVs were phased with N50 of phased block genomic span 751 bp. As for the PacBio data, the phased haplotypes span extended more genomic regions with a phase rate approaching 95%. While for Hi-C reads, although only 42% SNVs were phased, the assembled haplotypes stride across chromosomes, and the MVP block maintains AN50 of more than 6 Mbp. The hybrid phased haplotype significantly improved considering the AN50, with more than 98% of phased SNVs.

In addition, we also assembled haplotypes from a high-depth Chicago HiRise resequencing data for a single donkey jack (Willy). As shown in Table 2, we phased more than 2.5 million, 79% SNVs. The MVP block with the HiRise library demonstrated an adjusted span of more than 10 Mbp.

**TABLE 2:** Haplotype block statistics with diverse sequencing protocols for Willy.

	Adjusted N50/span^b^	N50/span	Phased SNVs	Phased SNVs (percent)
Chicago HiRise	458	34,623,120	2,026,095	79.24%
Chicago HiRise (MVP block)	10,504,902	20,919,015	21,290	NA
Population-assisted haplotype^a^	98,153,680	98,588,714	2,518,705	99.50%

a Population-assisted haplotype is a combination of sequencing phased haplotype and reference panel-based haplotype.

b AN50 is defined as N50 of adjusted span, which is the span of haplotype block times the proportion of phased SNP. Sequencing phased haplotype was phased based on merged sequencing data from multiple protocols: Illumina reads, PacBio, and Hi-C.

We built consensus sequences from r220 phased haplotypes. We constructed a pseudodiploid genome of *Equus asinu* for the first time (Xinyao, 2021). Pseudo-genome is available at https://doi.org/10.6084/m9.figshare.14222312.

### 3.2 Donkey Haplotype Dataset

We constructed an *Equus asinu* reference panel based on the variation data of 156 individual donkeys. Since Eagle v2.4.1 is mainly used for human data, we built a genetic map based on the reported genetic data of *Equus*. Unlike humans, donkeys have 30 pairs of chromosomes; thus, we optimized the calculation process. Combining the reported data of donkeys with whole-genome sequencing and our resequenced data, we constructed a haplotype dataset containing 156 individuals. The haplotype dataset contains 39,375,057 SNPs passed quality control filters, which are polymorphic across 156 samples.

**FIGURE 2:**
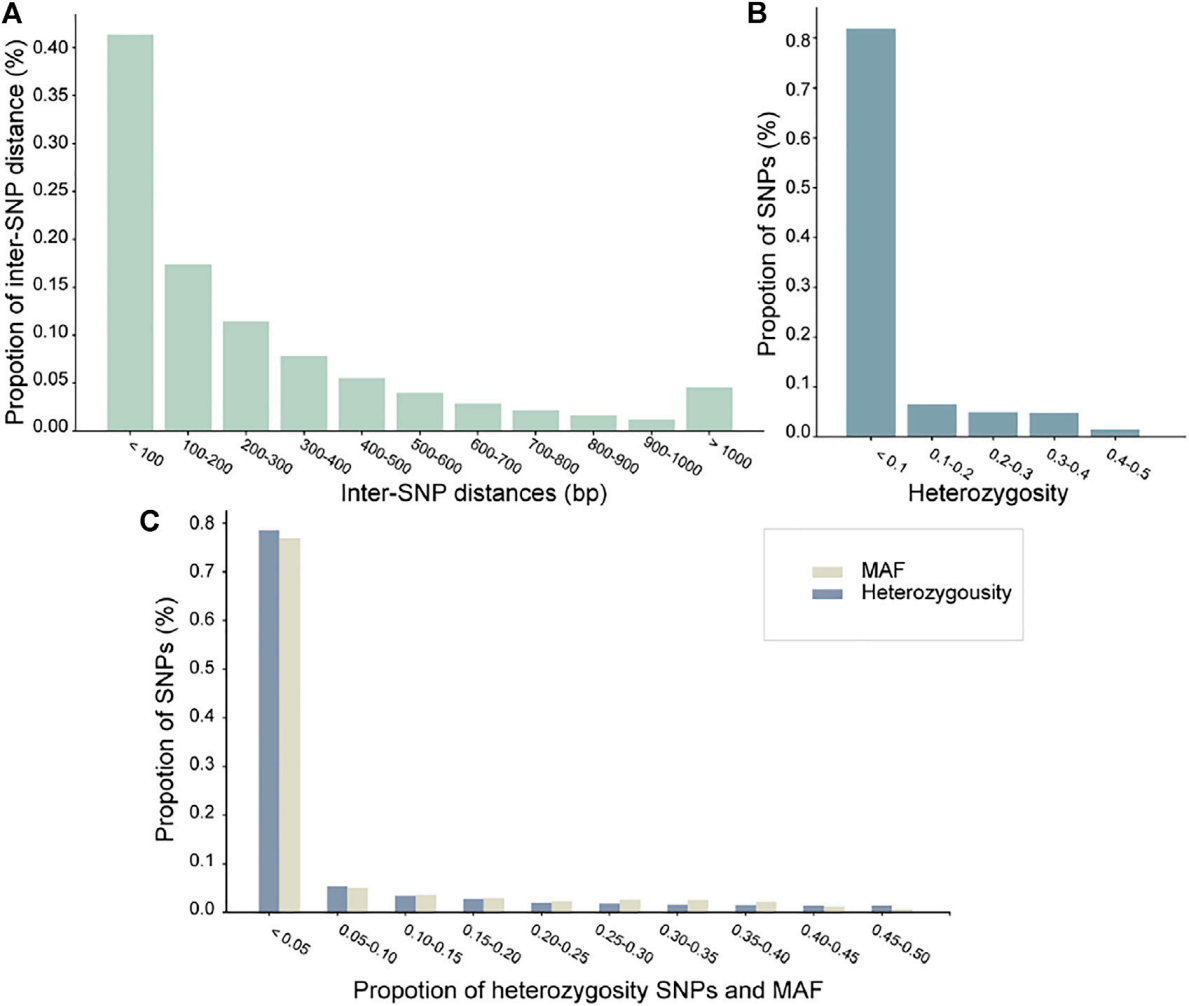
**(A-C)** are bar graphs to visualize SNP statistics in the donkey haplotype dataset. The horizontal axis represents the distances inter-SNPs, distribution of heterozygosity SNPs, and comparison of MAF and heterozygosity SNPs. The vertical axis represents the ratio of SNP/inter-SNPs (%).

Next, we performed statistics and analysis on the phased dataset (Figures 2A–C). Figure 2A indicated that 5% of inter-SNP distances are longer than 1 kb. Although most varying loci in the dataset are rare (∼10%), most heterozygous loci within an individual are due to shared SNPs, as displayed in Figure 1B. Minor allele frequency (MAF) and heterozygosity of SNPs maintained the same trend; rare alleles achieved a ∼80% detection rate, which indicates that rare alleles can be accurately genotyped (Figure 2C).

**FIGURE 3:**
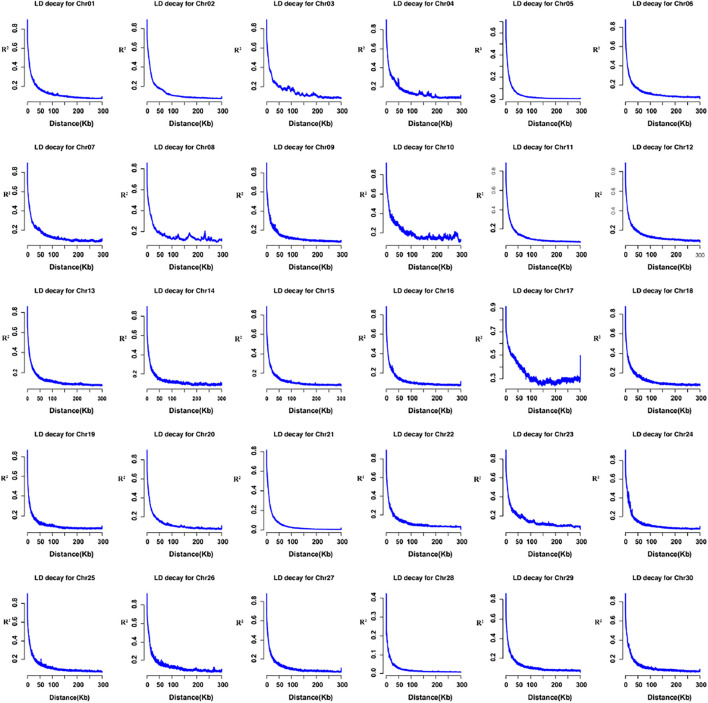
LD decay by chromosome (1-30); the horizontal axis represents the distance (kb), the y-axis shows the pair-wise r^2^ values.

LD is an allelic association along a chromosome and carries a set of specific alleles. Here, we selected three genes for LD demonstration in Supplementary Figures S1A–C, *KITLG*, *TPE1*, and *TBX3* reported in previous donkeys’ research. Then we estimated population LD decay for each chromosome. The LD decay distance of the domestic donkey was around 4.1 kb (r^2^ = 0.5), as suggested in Supplementary Figure S2. The per-chromosome statistics is shown in Figure 3, ranging from 1.5 to 9.4 kb (r^2^ = 0.5).

**FIGURE 4:**
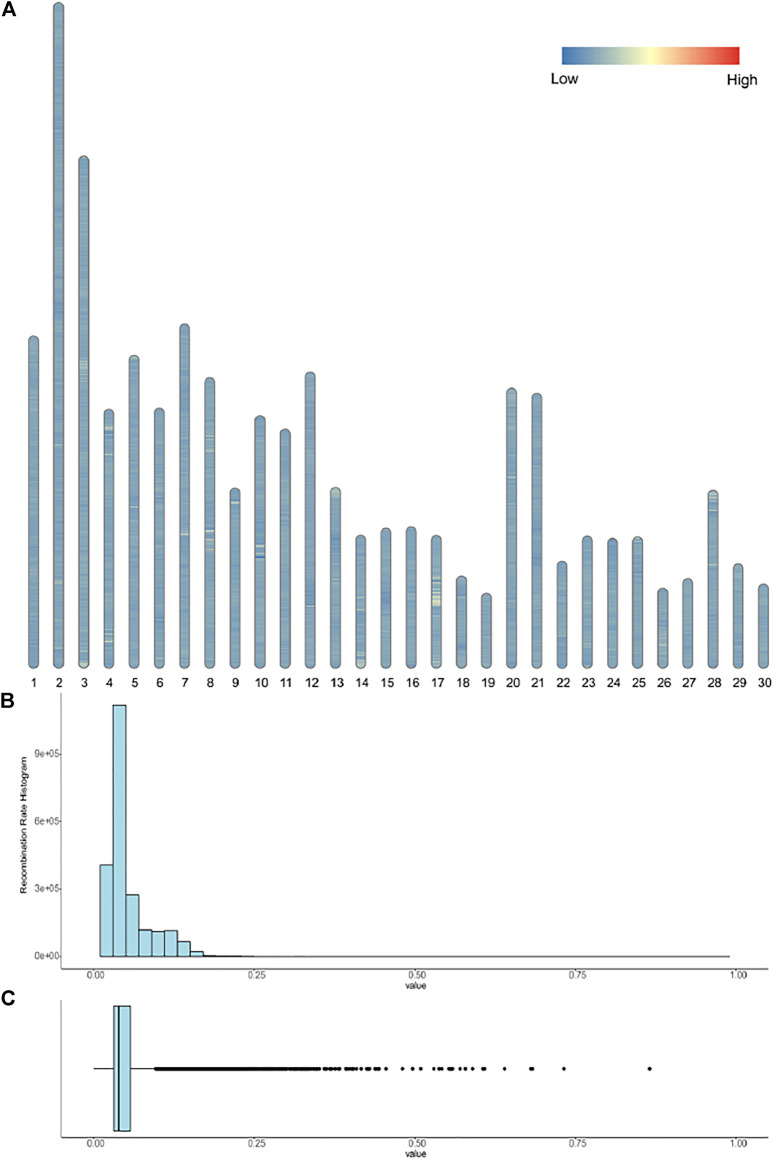
**(A)** show the recombination events among the 30 chromosomes; segments with a recombination rate greater than 0.3 are marked red. **(B-C)** are the histogram and box plot of the recombination rates; the x-axis represents the recombination rates.

We calculated the recombination rates among the whole donkey haplotype dataset. In Figures 4A–C, we presented the distribution of recombination events on the entire donkey genome. The average recombination rate is around 5% within a 1 kb window size, suggesting recombination events frequently occur in donkey domestication and isolation. In Supplementary Figures S3A–C, we calculated the recombination rates within the 0.1 kb window size in four genes, similar to Supplementary Figure S2. The results demonstrated that the recombination rate shares the same patterns as the LD value.

### 3.3 Integrating Read-Based and Population-Based Phasing

We used an in-house script to integrate read-based and population-based haplotypes for r220 and Willy (Table 1). After integrating, the phased rate for r220 and Willy increased to 99.73 and 99.50%, respectively. We reported a hybrid assembled haplotype for r220 based on diverse sequencing data adopted here.

**FIGURE 5:**
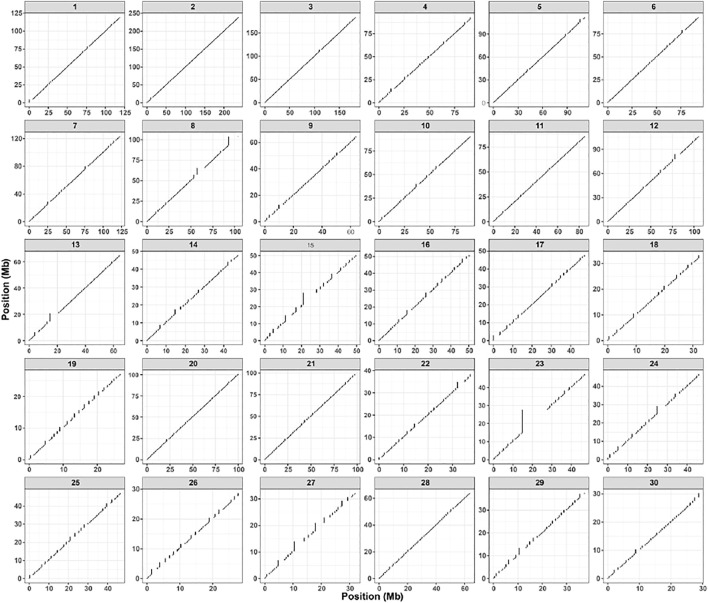
Figures show the genomic span for an individual donkey among each autosome (1-30); the x- and y-axis display the chromosome-level phase set position(Mb).

As represented in Figure 5, we reconstructed the complete chromosomal-scaled-haplotype of r220 with completeness, based on the integration of read-based and population-based phasing. Moreover, one haplotype block for each chromosome is compared with our hybrid assembled haplotype, suggesting that we achieved a complete-chromosome haplotype.

**FIGURE 6:**
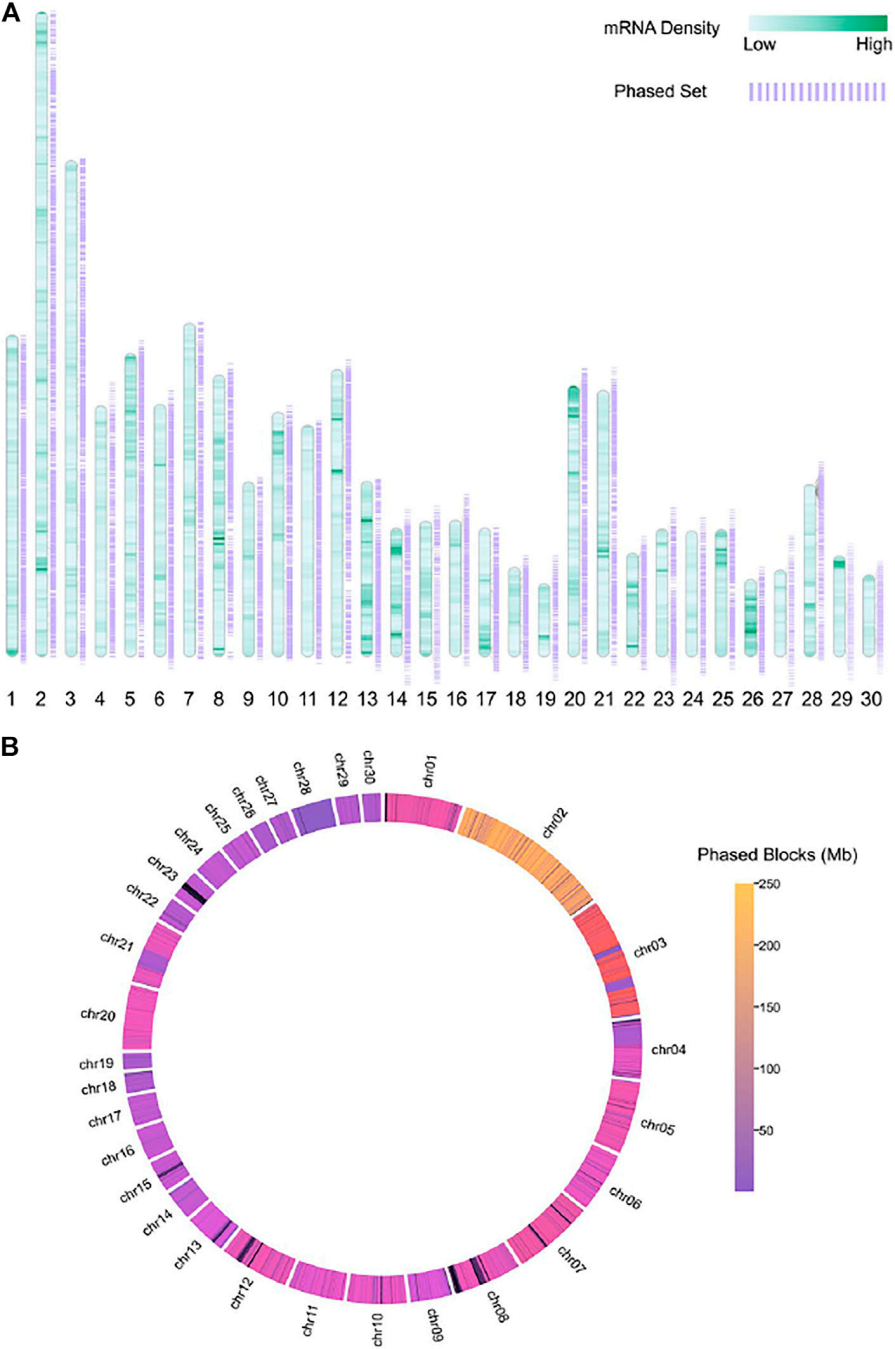
Figures show the density of mRNA and the chromosome-level phase set for a single individual donkey, different colors show the **(A)** mRNA density and **(B)** phased block(Mb).

We demonstrated the genomic span of the chromosome-level phase set on autosomes in Figure 6B. We also visualized the transcriptome of the donkey r220 in Figure 6A. The results suggested that the donkey genome is unphased in some coding regions due to repeats or complex structural variations. In addition, we phased heterozygous SNVs, which might be the reason for the phase set discontinuity.

### 3.4 Estimation of *Ne* According to Phased/Unphased Individual Donkey

**FIGURE 7:**
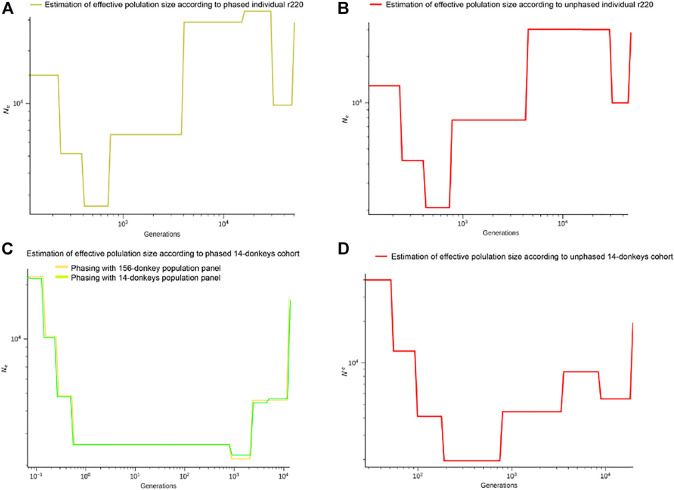
The dots indicated the admixture events on the SMC++ effective population size curves, **(A)** for phased individual donkeys (r220), **(B)** for un-phased individual donkeys, **(C)** for phased 14-donkeys cohort with 14/156-donkeys cohort, **(D)** for un-phased 14-donkeys cohort.

We performed SMC++ experiments on both phased and unphased data. As demonstrated in Figures 7A,B, both datasets shared a similar pattern of effective population size. We identified differences in generations with different *Ne*. In generations between 10^3^ and 10^4^, the estimated effective population size is smaller with phased data. In addition, the estimated effective population size demonstrated a fluctuation after generation 10^4^ for phased data, which is not recognized with unphased one. SMC++ with phased data displayed higher resolution for historical effective population size in more ancient time slots, suggesting haplotype phasing on the inference of population history.

In Figures 7A–C, we confirmed that the pattern of the effective population size presented by the 14 phased donkey groups is consistent with those provided by the phased r220 and is slightly different from the unphased r220. In Figure 7D, we found that 14 unphased donkeys have significant differences from other results in the trend of effective population size. In addition, we further explored the effect of different reference panel sizes on population phasing, and the results are shown in Figure 7C. Although the general trend is the same, we found that the effective population sizes after phasing have slight differences. Based on the reference panel of 156 donkeys provided by us, the results may be closer to the natural trend of the effective population size.

The results indicated that sequenced individuals’ accurate phasing results could provide a more detailed and precise downstream analysis, especially for non-model organisms. We believe a well-assembled haplotype genome and a haplotype database of donkey genome can assist the following donkey research.

## 4 Discussion and Conclusion

We assembled a hybrid single individual chromosome-scale haplotype of Dezhou donkey based on the diverse sequencing data from PacBio long-read Sequencing, Illumina short-read sequencing, Hi-C, and *Equus asinu* population reference panel. The AN50 was 93,063,389. The percentage of phased SNVs was 99.73%, indicating the hybrid phasing with multiple protocols and introducing a population reference panel boosted the continuity of the haplotype. In addition, we further phased 156 donkeys according to the reference panel, which demonstrated the potential to combine the result of single individual phasing and reference panel-based phasing with our high-depth samples.

*Equus asinu*, one of the *Equidae* family members, has a history of domestication for seven thousand years and has played an essential role in human civilization’s agricultural and transportation development (Geiger and Hovorka, 2015). The donkey still serves as a draught resource in some underdeveloped areas and is treated as a food source. Also, the donkey, one of the few species that can generate hybrid offspring with closely related species, provides insight into the analysis of allele-specific gene expression and alternative splicing (Wang et al., 2019).

As an extension of the genome, haplotypes contain allelic associations, evolutionary processes, gene flow, and population demography. In 2007, the Phase II human HapMap project reported more than 3.1 million SNPs and generality of recombination events (International HapMap Consortium, 2005; Frazer et al., 2007). In 2010, the 1000 genomes project provided a more detailed map of human genome variation, which presented the value dataset and tools for the human genome (Genomes Project et al., 2010). Many recent studies have focused on human haplotypes: 1) Neanderthal haplotype on chromosome 12 might be protective against severe disease, COVID-19, reducing ∼22% relative risk (Zeberg and Pääbo, 2021); 2) high-depth African haplotypes indicated the history of human migration and the demography of disease (Choudhury et al., 2020); 3) in the field of human immunology, phasing/haplotype information might be potential for polymorphic immunoglobulin heavy chain locus, and V, D, and J germline genes (Omer et al., 2020; Rodriguez et al., 2020). For non-human species, research mainly focused on model organisms and developing genomic and laboratory resources. Recently, modern resistance breeding involved haplotype-based approaches for crop production (Ogawa et al., 2018; Brinton et al., 2020; Liu et al., 2020). Haplotypes were potential indicators for fertility, domestication, and adequate population size (Moradi et al., 2017; Xu et al., 2019). Additionally, some studies applied trio-phase for interspecies (F1 hybrid) for comparative genomics and reconstructing the population admixture (Rice et al., 2020).

Although the haplotype information has been widely examined in *Homo sapiens*, analysis of many non-model organisms is still based on independent SNPs. However, the haplotype information provides significant insights into conservation genomics and the study of biodiversity (Leitwein et al., 2020). LD patterns across the genome were adopted in demographic inference and research of historical gene flow at both within-population and between-population levels (Duranton et al., 2018; Schumer et al., 2018).

In addition, phasing errors dramatically influence the inferred demographic history in demographic estimation tools like MSMC. SMC++ provides consensus and accurate results by human sequencing data with different phasing switch error rates and unphased data. However, in our case, the demographic estimation results by SMC++ from phased data and unphased data in the donkey population are different. The haplotype information also suggests that the result of genetic admixture and hybridization that might further contribute to establishing a conservation strategy (Allendorf et al., 2010; Harris and Nielsen, 2016; Leitwein et al., 2018).

We described the hybrid single individual assembled haplotype of the Dezhou donkey based on multiple protocols’ high depth sequencing data. We also constructed a donkey reference panel based on the cohort phasing of our resequenced 156 donkey individuals. We further phased our 156 donkeys according to the reference panel. In addition, we demonstrated the potential to combine single individual phasing and reference panel-based phasing with our high-depth samples. The pseudodiploid genome was created based on the mixed results.

Algorithm 1: Haplotype merging algorithm.

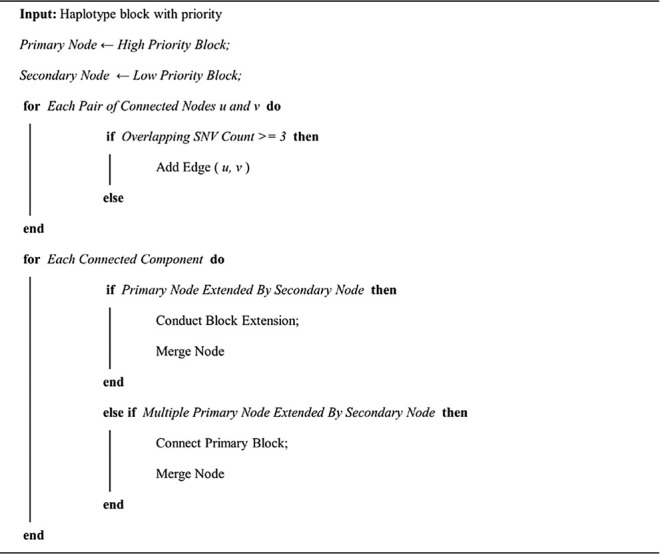

## Data Availability

The single individual sequencing data that support the findings of this study are available in Genebank (SRS3193390) and ENA(PRJEB24845). The population sequencing data that support the findings are available under the accession KT896508-KT896510, SRR1562345, ERR650932-ERR654612, ERR669419-ERR669469, ERR650540-ERR650547, ERR650570-ERR650703, and PRJCA001131. The source code of SpecHap is available at https://github.com/deepomicslab/SpecHap. Pseudo-genome is available at https://doi.org/10.6084/m9.figshare.14222312.
