# Supplementary figures and images for: Chromosome-Level Haplotype Assembly for Equus asinu
Source: Front Genet. 2022 May 27;13:738105. doi: 10.3389/fgene.2022.738105 (PMC9186339; doi:10.3389/fgene.2022.738105)

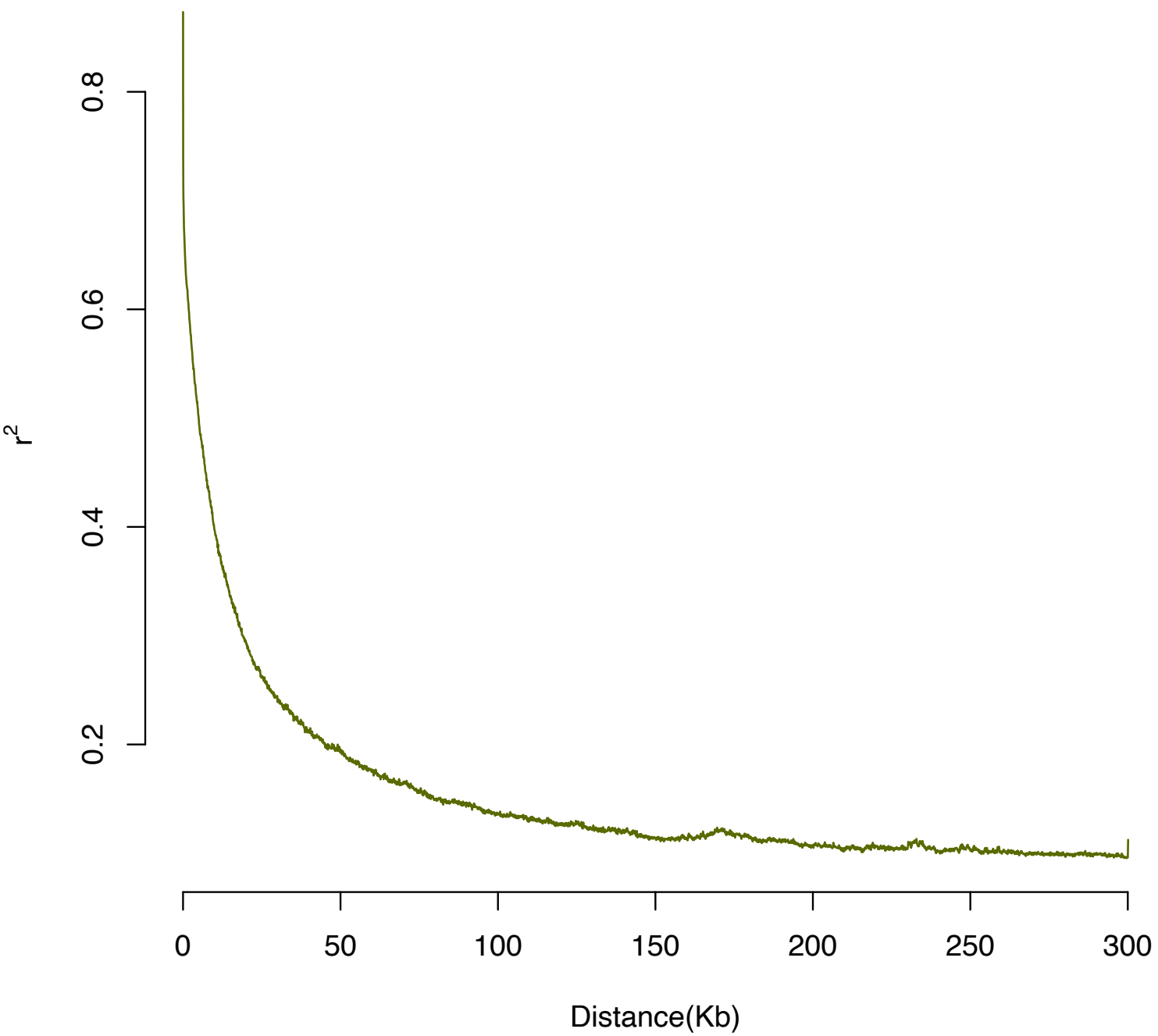

Supplement: Supplementary file 1 [file Image2.pdf]

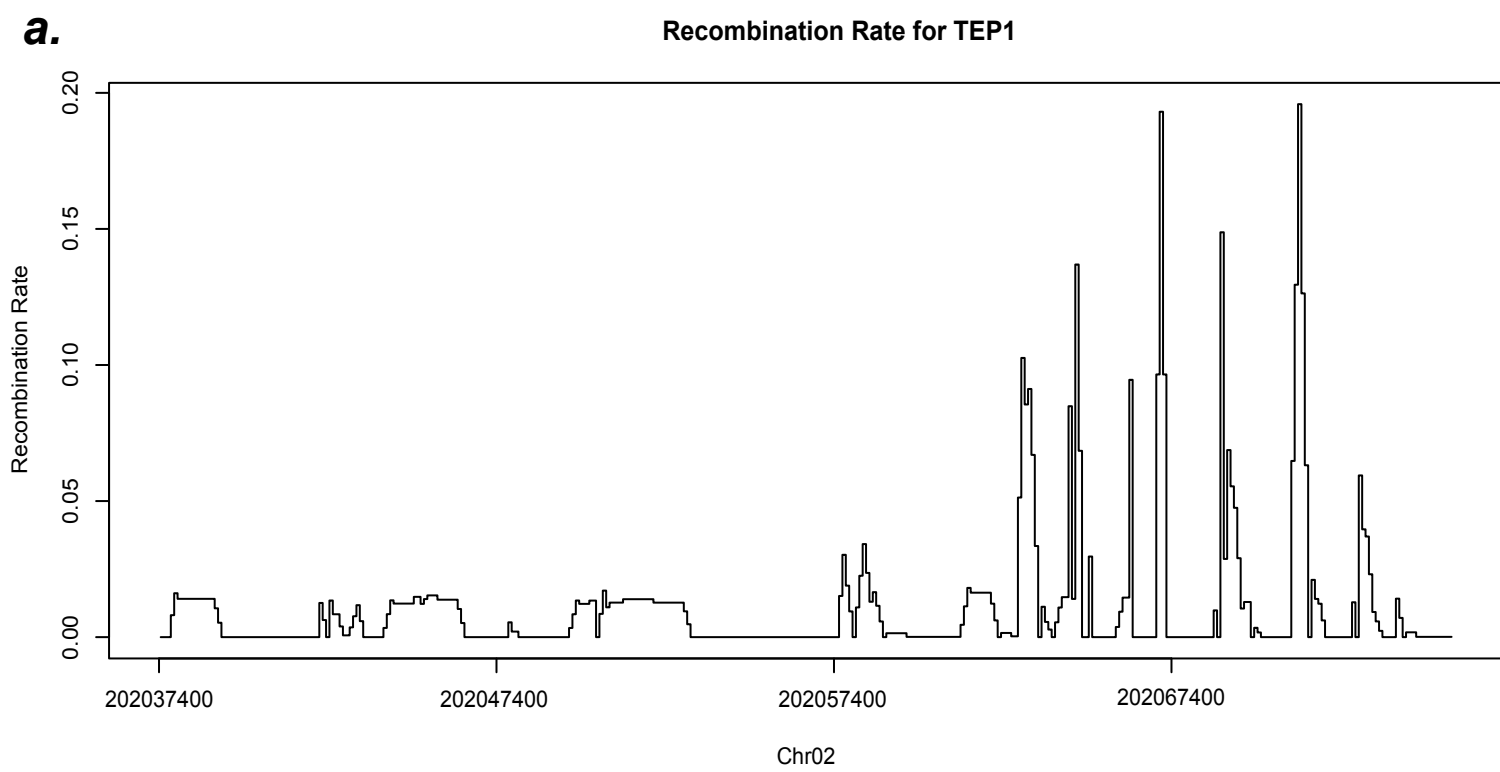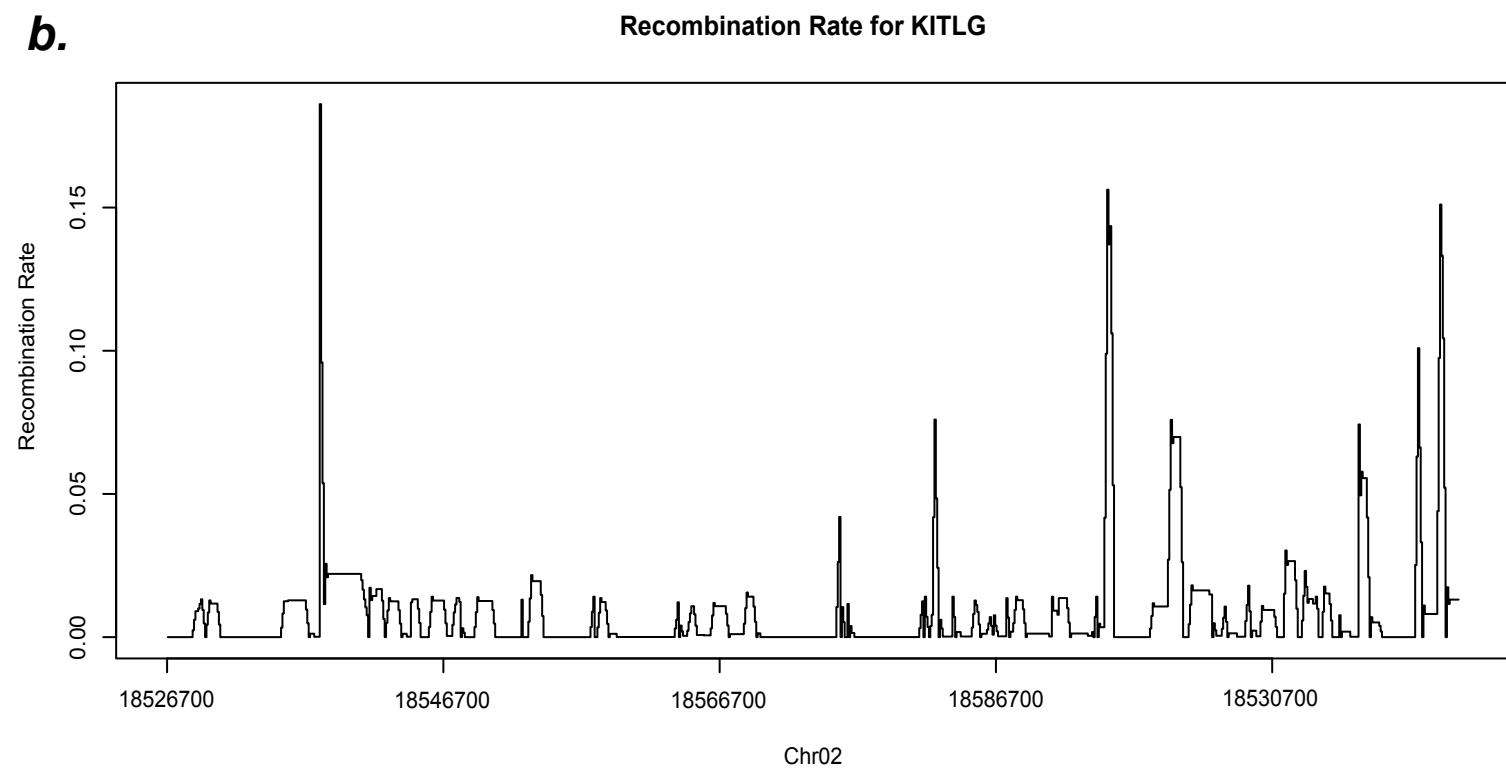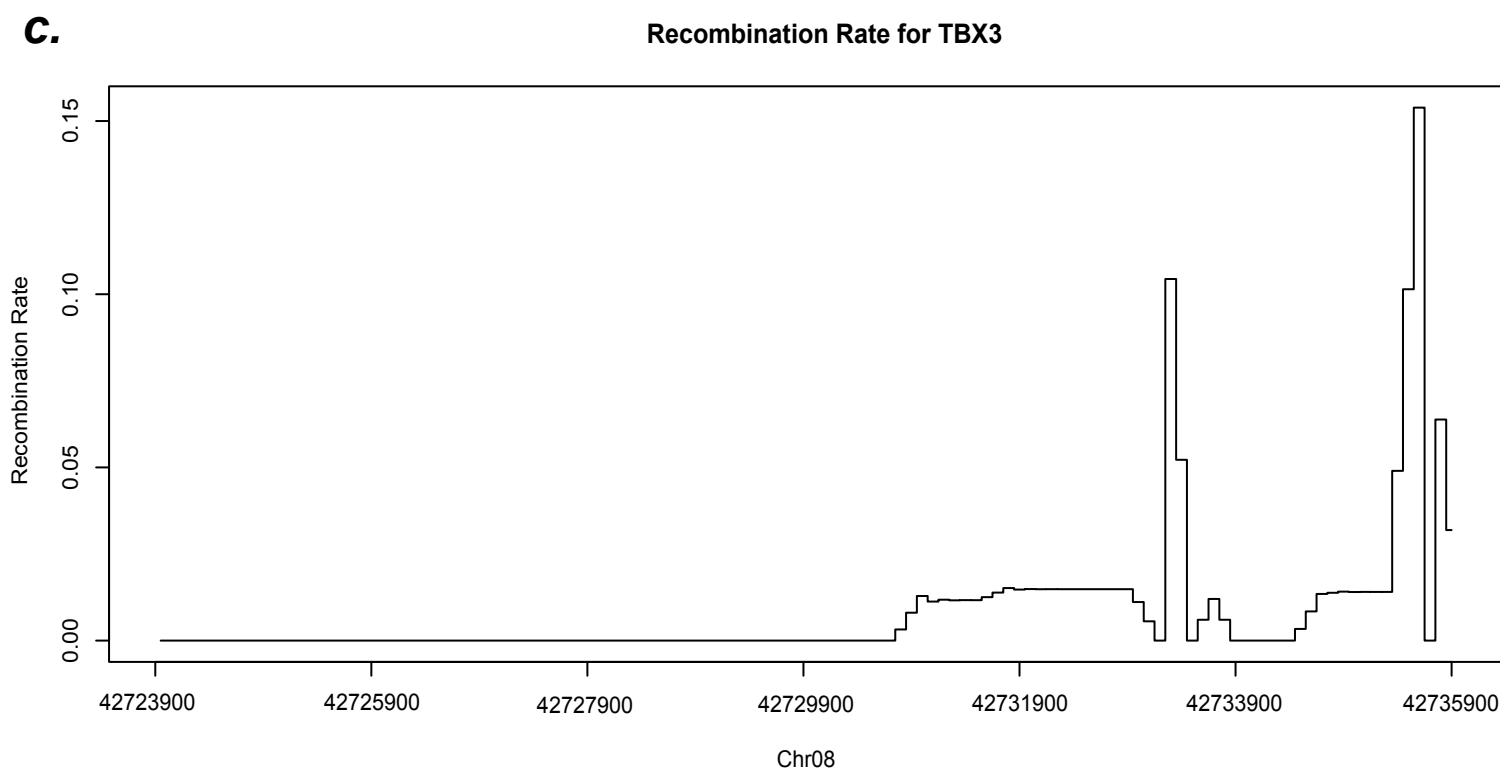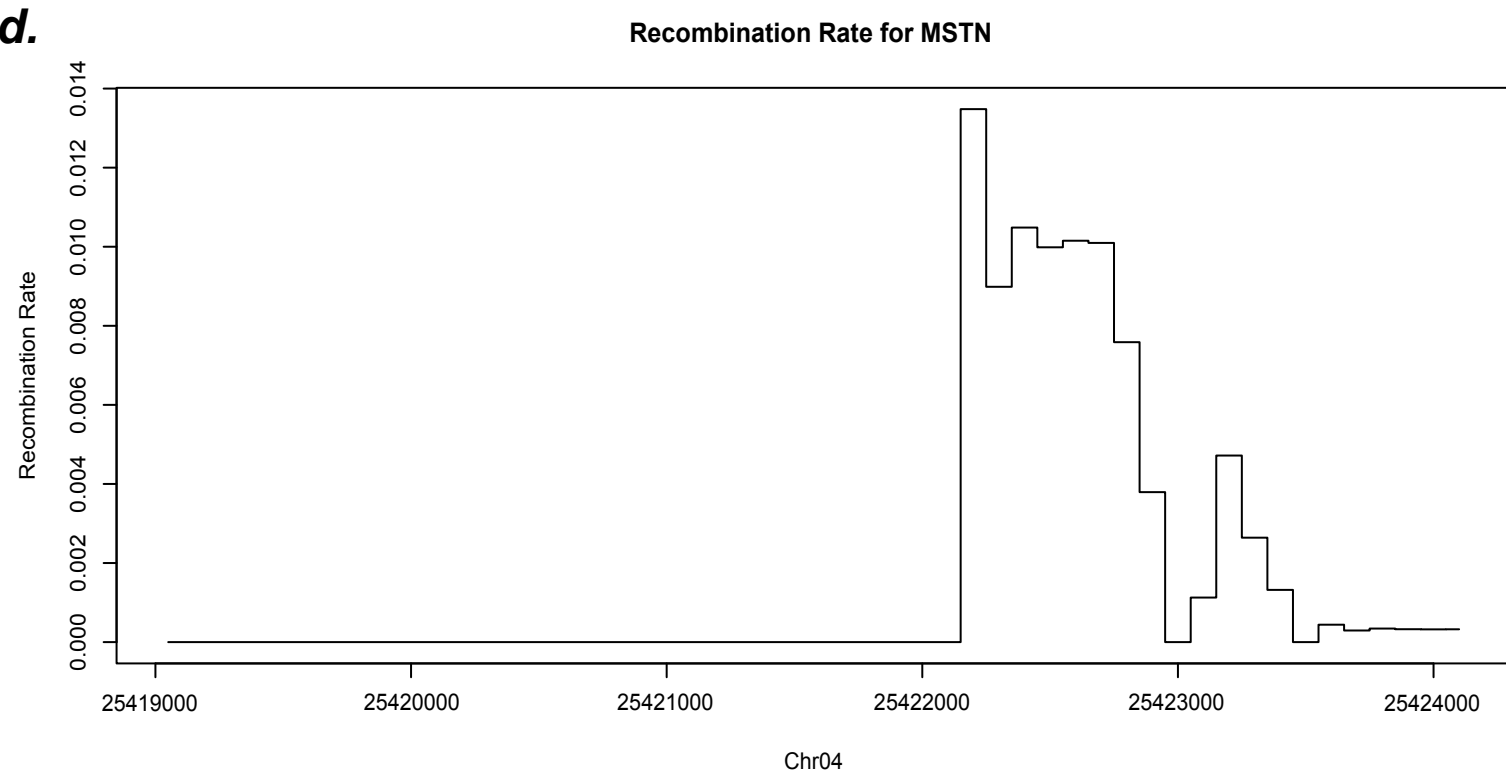

Supplement: Supplementary file 2 [file Image3.pdf]
