# Supplementary material for: Chromosome-Level Haplotype Assembly for Equus asinu
Source: Front Genet. 2022 May 27;13:738105. doi: 10.3389/fgene.2022.738105 (PMC9186339; doi:10.3389/fgene.2022.738105)
Supplement: Supplementary file 3 [file Image1.pdf]

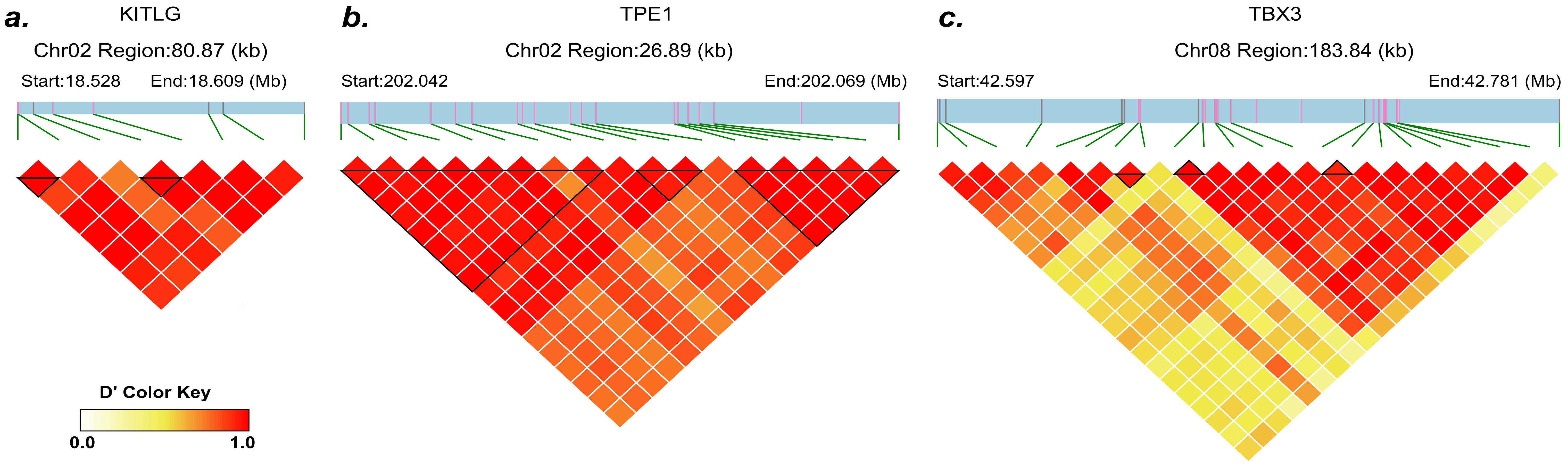
a.
KITLG
Chr02 Region:80.87 (kb)
Start:18.528
End:18.609 (Mb)
b.
TPE1
Chr02 Region:26.89 (kb)
Start:202.042
End:202.069 (Mb)
c.
TBX3
Chr08 Region:183.84 (kb)
Start:42.597
End:42.781 (Mb)
D' Color Key
0.0
1.0
